# Urinary Tract Infections in a Single-Center Bulgarian Hospital: Trends in Etiology, Antibiotic Resistance, and the Impact of the COVID-19 Pandemic (2017–2022)

**DOI:** 10.3390/antibiotics14100982

**Published:** 2025-09-30

**Authors:** Milena Yancheva Rupcheva, Kostadin Kostadinov, Yordan Kalchev, Petya Gardzheva, Eli Hristozova, Zoya Rachkovska, Gergana Lengerova, Andreana Angelova, Marianna Murdjeva, Michael M. Petrov

**Affiliations:** 1 Department of Medical Microbiology and Immunology “Prof. Dr. Elissay Yanev”, Faculty of Medicine, Medical University of Plovdiv, 4002 Plovdiv, Bulgaria; milena.rupcheva@mu-plovdiv.bg (M.Y.R.); yordan.kalchev@mu-plovdiv.bg (Y.K.); petya.gardzheva@mu-plovdiv.bg (P.G.); eli.hristozova@mu-plovdiv.bg (E.H.); zoya.rachkovska@mu-plovdiv.bg (Z.R.); gergana.lengerova@mu-plovdiv.bg (G.L.); andreana.angelova@mu-plovdiv.bg (A.A.); mmurdjeva@yahoo.com (M.M.); 2 Research Institute at Medical University—Plovdiv (RIMU), 4002 Plovdiv, Bulgaria; kostadinr.kostadinov@mu-plovdiv.bg; 3 Laboratory of Microbiology, University Hospital St. George, 4002 Plovdiv, Bulgaria; 4 Department of Social Medicine and Public Health, Faculty of Public Health, Medical University of Plovdiv, 4002 Plovdiv, Bulgaria; 5 Department of Microbiology and Virology, Faculty of Pharmacy, Medical University of Pleven, 5800 Pleven, Bulgaria; 6 Institute for Innovation and Smart Technology (IIST), University of Telecommunication and Posts, 1700 Sofia, Bulgaria

**Keywords:** urinary tract infections, uropathogens, antimicrobial resistance, cystitis, pyelonephritis, COVID-19, urinalysis

## Abstract

**Background**: Urinary tract infections (UTIs) are among the most common hospital- and community-acquired infections, creating a substantial healthcare burden due to recurrence, complications, and rising antimicrobial resistance. Accurate diagnosis and timely antimicrobial therapy are essential. This study aimed to identify trends in the etiology, treatment, and resistance patterns of UTIs through a retrospective analysis of urine isolates processed at the Laboratory of Microbiology at University Hospital St. George in Plovdiv, the largest tertiary care and reference microbiology center in Bulgaria, between 2017 and 2022. **Materials and Methods**: A retrospective single-center study was performed at the hospital’s Microbiology Laboratory. During the study period, 74,417 urine samples from 25,087 hospitalized patients were screened with the HB&L UROQUATTRO system. Positive specimens were cultured on blood agar, Eosin-Methylene Blue, and chromogenic media. Identification was performed using biochemical assays, MALDI-TOF MS, and the Vitek 2 Compact system. Antimicrobial susceptibility testing included disk diffusion, MIC determination, broth microdilution (for colistin), and Vitek 2 Compact, interpreted according to EUCAST standards. Descriptive analysis and temporal resistance trends were evaluated with regression models, and interrupted time-series analysis was applied to assess COVID-19-related effects. **Results**: Out of 10,177 isolates, Gram-negative bacteria predominated (73%), with *Escherichia coli*, *Klebsiella pneumoniae*, and *Proteus mirabilis* as the leading pathogens. Among Gram-positives, *Enterococcus faecalis* was the most frequent. In the post-COVID-19 period, ESBL production increased in *E. coli* (34–38%), *K. pneumoniae* (66–77%), and *P. mirabilis* (13.5–24%). Carbapenem resistance rose in *K. pneumoniae* (to 40.6%) and *P. aeruginosa* (to 24%), while none was detected in *E. coli*. Colistin resistance increased in *K. pneumoniae* but remained absent in *E. coli* and *P. aeruginosa*. High-level aminoglycoside resistance in *E. faecalis* was stable (~70%), and vancomycin resistance in *E. faecium* rose from 4.6% to 8.9%. **Conclusions**: Both community- and hospital-acquired UTIs in Southeastern Bulgaria are increasingly linked to multidrug-resistant pathogens, particularly ESBL-producing and carbapenem-resistant *Enterobacterales*. Findings from the region’s largest referral center highlight the urgent need for continuous surveillance, rational antibiotic use, and novel therapeutic approaches.

## 1. Introduction

Urinary tract infections can involve any part of the urinary tract and are most commonly caused by various types of bacteria [1]. UTIs affect all age groups but show bimodal peaks: young sexually active women and elderly patients with comorbidities or indwelling urinary catheter use. Risk factors include diabetes, urinary catheters, urinary tract abnormalities, and prior antibiotic exposure [2,3].

UTIs are among the most common bacterial infections acquired in both the community and hospitals [4]. They rank among the most frequent infections in clinical practice worldwide, and their incidence and severity are probably even higher than the available data, as they are not subject to mandatory reporting [5]. According to a study in the United States, UTIs are responsible for nearly 7 million visits to the doctor’s office and 1 million visits to the emergency department, which are accompanied by 100,000 hospitalizations per year [6]. According to *Thomas P. Lodise*, medical costs for a 30-day treatment of newly diagnosed acute complicated urinary tract infections amounted to USD 7.6 billion in the United States for the period 2016–2019 [7]. Globally, the incidence of UTIs increased by 60% in 2019 compared to 1990. The mortality rate from UTIs was found to be 3.13% per 100,000 in 2019 [6]. Urinary tract infections are among the most common causes of sepsis, which is one of the most severe and life-threatening infectious complications [8]. Urosepsis accounts for approximately 5 to 7% of all cases of severe sepsis in hospitals [9].

Urinary tract infections are generally classified based on their anatomical location (lower cystitis and upper pyelonephritis) within the urinary system, the presence of complicating factors (uncomplicated and complicated), and whether symptoms are present or absent (asymptomatic and symptomatic) [10,11]. According to the guidelines of the European Association of Urology, uncomplicated cystitis refers to a lower urinary tract infection (LUTI) in men or non-pregnant women who are otherwise healthy and without underlying comorbidities. In contrast, complicated cystitis is associated with risk factors that increase the likelihood and severity of infection or the risk of failure of antibiotic therapy [12].

Acute pyelonephritis is defined as a severe form of urinary tract infection affecting the renal pelvis and calyceal system, with symptoms ranging from mild discomfort to life-threatening illness or death [13]. Pyelonephritis is often associated with high levels of antimicrobial resistance among its causative pathogens [11]. According to the Bulgarian Association of Microbiologists (BAM), the differentiation between cystitis and pyelonephritis is based on clinical symptoms, physical examination findings, and laboratory data [14].

Asymptomatic bacteriuria is not considered a urinary tract infection in the absence of symptoms [15]. It should be differentiated from both uncomplicated and complicated UTIs, as it is viewed more as commensal colonization that does not require antibiotic treatment [16].

According to the Public Health England guidelines, urine culture is usually deemed unnecessary in women presenting with two or three typical UTI symptoms, and empirical antibiotic treatment is recommended without further testing [17]. However, establishing a diagnosis of symptomatic UTIs requires careful clinical evaluation and laboratory confirmation through urinalysis and microbiological urine culture [18].

Diagnosis and management depend on patient-specific factors and the extent of the disease. Urinary tract infection diagnosis relies not only on clinical symptoms but also on the results of clinical, laboratory, and microbiological examinations.

Traditional microbiological methods for the diagnosis of UTIs are based primarily on urine culture, followed by biochemical identification of the isolates using both conventional and automated systems such as Vitek 2 Compact. In addition, modern technologies such as MALDI-TOF MS are increasingly being used to achieve rapid and precise identification of isolated microorganisms. Automated screening methods such as turbidimetry (HB&L Uroquattro, Alifax) can also be used for early screening of negative urine samples, although this approach is not routinely applied in all laboratories. Antimicrobial susceptibility testing is usually performed using the DDM and, when necessary, by determining the MIC. Although these conventional approaches remain the gold standard of routine microbiological diagnostics, they have some limitations in terms of the time required to obtain results. For this reason, the introduction of modern molecular genetic methods, including multiplex real-time PCR and MALDI-TOF MS, has significantly improved the speed and accuracy of etiological diagnosis in clinical practice.

Rapid and accurate microbiological diagnostics are relevant for reducing antimicrobial drug resistance. Thanks to modern molecular genetic methods—multiplex real-time PCR (real-time mPCR) and the new technology of matrix-assisted laser desorption ionization time of flight on the principle of mass spectrometry (MALDI-TOF), with the Vitek MS device—an opportunity for accelerated and accurate etiological diagnostics has been created.

Antimicrobial resistance patterns vary substantially between countries, regions, and even hospitals. Local surveillance is essential for guiding empiric therapy and stewardship [19,20]. The growing challenge of antimicrobial resistance (AMR) further amplifies the burden of UTIs. The World Health Organization has identified antimicrobial resistance (AMR) as a leading global public health threat, with 1.27 million deaths reported in 2019 directly attributable to it [21]. In this context, the so-called ESKAPE pathogens—*Enterococcus faecium*, *Staphylococcus aureus*, *Klebsiella pneumoniae*, *Acinetobacter baumannii*, *Pseudomonas aeruginosa*, and *Enterobacter* spp.—dominate hospital-acquired infections and demonstrate multidrug resistance [22,23]. Some of these pathogens have also been prioritized in the updated WHO list of bacterial priority pathogens (2024), which addresses multidrug resistance and the need for measures to address AMR [24]. The isolation of multidrug-resistant microbial pathogens from urine is becoming more prevalent. This phenomenon incurs substantial economic consequences, encompassing both treatment expenses and several hospitalizations and stays [25]. In Europe, antimicrobial resistance (AMR) has been estimated to cause 33,000 fatalities each year, with urinary pathogens being significant contributors [26]. The COVID-19 pandemic linked inappropriate or excessive use of broad-spectrum antibiotics to accelerated AMR trends in uropathogens worldwide [18]. The COVID pandemic is believed to have disrupted efficient infection control practices, leading to problems with infection prevention and control measures and delays in antibiotic stewardship programs [27]. Reports also indicate a surge in the use of broad-spectrum antibiotics, particularly among hospitalized patients who do not have bacterial co-infection [28]. Evidence suggests that the pandemic has intensified resistance pressures due to the widespread empirical use of broad-spectrum antibiotics in patients without confirmed bacterial infection [12]. This has created uncertainty regarding the trajectory of resistance in urinary pathogens and reaffirmed the value of local surveillance [29]. AMR patterns vary substantially across regions and healthcare systems, and local epidemiological data are essential to guide effective empirical therapy, infection control, and stewardship programs [30].

By studying the etiological structure, it is possible to propose strategies to avoid irrational use of antibiotics, knowledge of the pathogens circulating in the local area, and the levels of their antimicrobial drug resistance. This would significantly contribute to preventing the emergence of local resistance types. The rapid microbiological results provide the prospect of targeted treatment and a more favorable outcome for the patient’s disease.

To address these gaps, we conducted a retrospective, single-center epidemiological study at the Microbiology Laboratory of University Hospital St. George in Plovdiv, Bulgaria—the largest tertiary care and reference microbiology facility in Southeastern Bulgaria. Our objectives were to (1) describe the etiological spectrum of UTIs among inpatients and outpatients over a six-year period (2017–2022); (2) assess antimicrobial resistance patterns across leading uropathogens; (3) evaluate temporal resistance trends using regression models; and (4) quantify the impact of the COVID-19 pandemic through interrupted time-series analysis.

## 2. Results

### 2.1. Temporal Distribution and Taxonomic Composition of Urinary Isolates (2017–2022)

Over the six-year period 2017–2022, the Laboratory of Microbiology at University Hospital St. George—Plovdiv, Bulgaria, received 74,417 urine samples from 25,087 hospitalized patients. A total of 10,177 microorganisms were isolated, and the overall isolation rate was 12%. The annual distribution is presented in Table 1.

Between 2017 and 2020, the number of isolates declined by approximately 32%. This trend reversed in 2021–2022, when counts increased sharply: by 68% in 2022 compared with 2020, and by 15% compared with 2017.

Among the isolated etiological agents for the study period, Gram-negative bacteria consistently dominated, with an average of 73% of isolates. In Gram-positive bacteria, it was relatively stable, with minor year-to-year fluctuations, while fungal isolates demonstrated a marked upward trend, rising from approximately 2% (n = 34) in 2017 to 6% (n = 114) in 2022.

In 2017, a total of 1711 microorganisms were isolated. Of these, 73% (n = 1251) were Gram-negative bacteria, mainly from the order *Enterobacterales*, followed by Gram-positive bacteria (25%; n = 426) and a significantly smaller relative share of fungi (2%; n = 34). By 2022, a significant increase of 6% in the relative share of isolated pathogenic fungi was observed.

### 2.2. Etiological Structure

**Figure 1:**
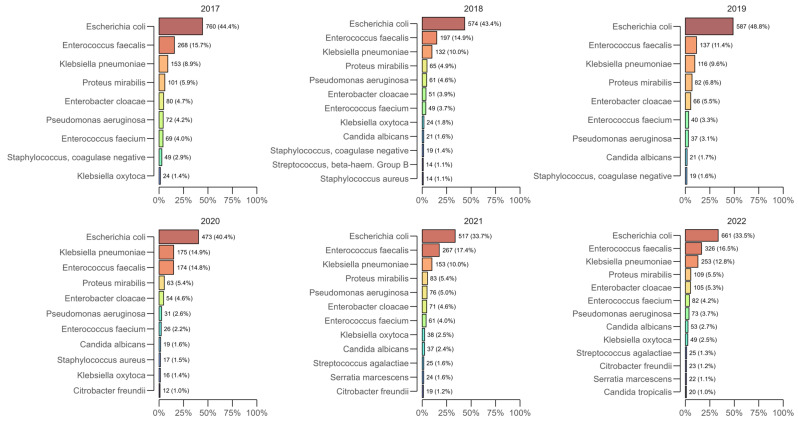
Distribution of urine isolates by year for the period 2017–2022.

Across all years of the study, the most commonly isolated bacterial species from positive urine cultures were *Escherichia coli*, *Enterococcus faecalis*, *Klebsiella pneumoniae*, and *Proteus mirabilis*, which together accounted for approximately two-thirds of the annual isolates (Figure 1). *E. coli* remained the leading pathogen each year, peaking at 48.8% (n = 587) in 2019 and subsequently decreasing to 33.5% (n = 661) in 2022, but consistently ranked first. *E. faecalis* generally ranked second among the most common isolates (e.g., 15.7% in 2017, 17.4% in 2021, and 16.5% in 2022), while *K. pneumoniae* showed a significant increase during the pandemic period—14.9% (n = 175) in 2020, remaining elevated in 2021 (10.0%) and 2022 (12.8%; n = 253) compared to 2017–2019. *P. mirabilis* was detected at an approximately constant 5–7% per year (e.g., 5.9% in 2017, 6.8% in 2019, and 5.5% in 2022).

Isolation of pathogenic fungi (yeasts) progressively increased during the study period. *Candida* spp. accounted for 2% (n = 34) of isolates in 2017 and 3% in 2018–2020 (n = 37 in 2018, n = 30 in 2019, and n = 31 in 2020), increased to 4% (n = 69) in 2021, and reached 6% (n = 114) in 2022. *C. albicans* was the predominant species (1% in 2017; 1.6 to 2.7% in 2018–2022), with increasing numbers of *Candida non-albicans* species isolated towards the end of the period as *C. glabrata*, *C. kefyr*, and *C. lusitaniae* emerged in 2022. These data indicate a significant increase in *Candida* spp., likely due to the need for intensive care during hospital treatment and the use of antimicrobial agents during the pandemic era.

### 2.3. Temporal Trends in Antimicrobial Resistance

**Figure 2:**
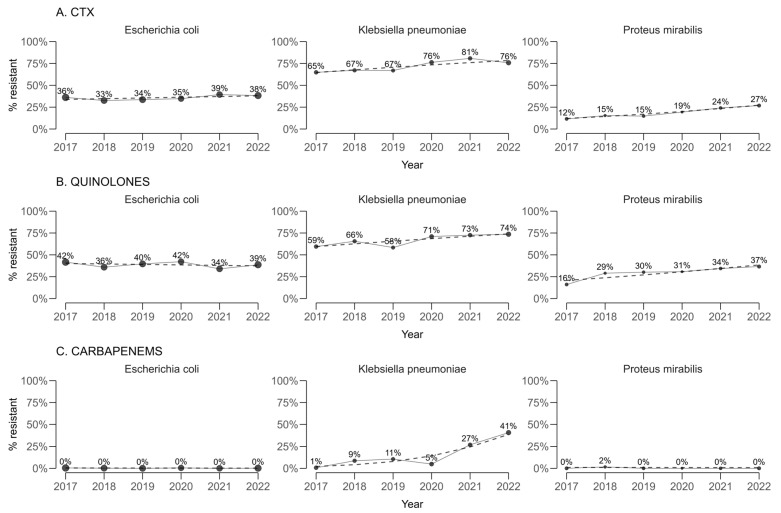
Temporal trends in (**A**) cefotaxime (proxy for ESBL production), (**B**) quinolone, and (**C**) Carbapenems resistance among *Escherichia coli*, *Klebsiella pneumoniae*, and *Proteus mirabilis* (2017–2022).

For cefotaxime, used as a proxy for extended-spectrum beta-lactamase (ESBL) production (Figure 2), statistically significant annual increases in resistance proportions were estimated for *Klebsiella pneumoniae* (+2.77 percentage points per year; 95% CI 2.66 to 2.88) and *Proteus mirabilis* (+3.11 percentage points per year; 95% CI 3.05 to 3.16). A smaller but still statistically significant increase was observed for *Escherichia coli* (+0.823 percentage points per year; 95% CI 0.789 to 0.858).

For quinolones, resistance in *K. pneumoniae* and *P. mirabilis* also increased significantly over time, with annual changes of +2.93 percentage points (95% CI 2.85 to 3.00) and +3.62 percentage points (95% CI 3.49 to 3.74), respectively. In contrast, *E. coli* exhibited a statistically significant decreasing trend of −0.542 percentage points per year (95% CI −0.577 to −0.506).

When carbapenem resistance was assessed by pooling imipenem and meropenem, a marked and highly significant upward trend was identified in *K. pneumoniae* (+8.88 percentage points per year; 95% CI 8.60 to 9.15). Statistically significant but small decreases were observed for *E. coli* (−0.04 percentage points per year; 95% CI −0.03 to −0.05) and *P. mirabilis* (−0.06 percentage points per year; 95% CI −0.08 to −0.04).

**Figure 3:**
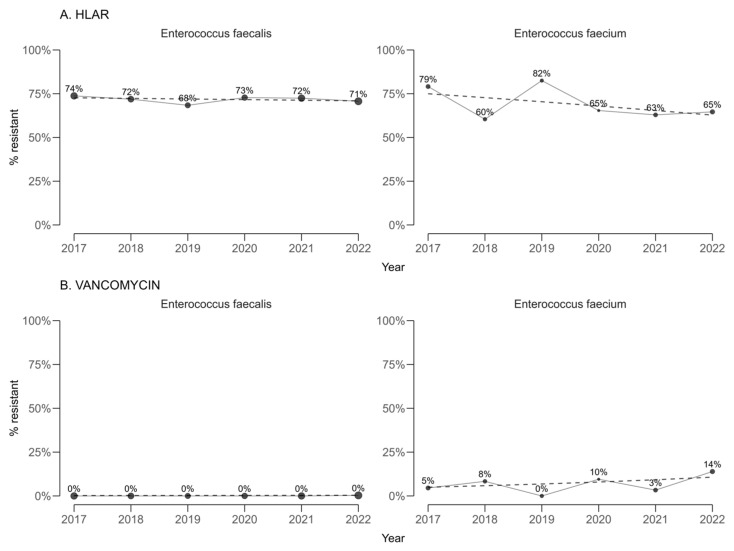
Temporal trends in (**A**) high-level gentamicin resistance (HLAR) and (**B**) vancomycin resistance among *Enterococcus faecalis* and *Enterococcus faecium* (2017–2022).

High-level gentamicin resistance (HLAR) among enterococci (Figure 3) showed statistically significant annual decreases in both *E. faecalis* (−0.35 percentage points per year; 95% CI −0.39 to −0.31) and *E. faecium* (−2.47 percentage points per year; 95% CI −2.86 to −2.08). Vancomycin resistance remained infrequent, and no statistically significant temporal trends were detected in either *E. faecalis* or *E. faecium*.

A before-and-after comparison between the earliest and latest study years confirmed these patterns. For cefotaxime, significant absolute increases in resistance were observed in *K. pneumoniae* (from 64.9% to 75.8%, *p* = 0.029) and *P. mirabilis* (from 11.6% to 26.9%, *p* = 0.019), with no significant changes for *E. coli*. Carbapenem resistance in *K. pneumoniae* rose dramatically from 1.36% to 40.4% (*p* < 0.0001). Quinolone resistance increased in *K. pneumoniae* from 59.5% to 73.6% (*p* < 0.0001) and in *P. mirabilis* from 16.1% to 36.6% (*p* < 0.0001).

### 2.4. COVID-19 Impact Analysis

COVID-period effects were evaluated using an interrupted time-series approach with beta regression, modeling resistance proportions from 2017 to 2019 to generate a counterfactual scenario representing the expected trajectory in the absence of COVID. Observed values for 2020–2022 were compared against these predictions to estimate absolute COVID-period effects in percentage points (pp).

**Figure 4:**
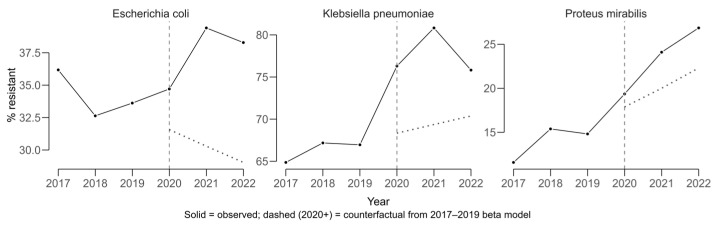
COVID-period effect analysis in trends for cefotaxime resistance (ESBL proxy) among *Escherichia coli*, *Klebsiella pneumoniae*, and *Proteus mirabilis* (2017–2022).

*Klebsiella pneumoniae* displayed a sustained upward trajectory in cefotaxime resistance, which is used as a proxy for ESBL production, prior to 2020 and exceeded its expected values during the COVID period (Figure 4). The average COVID-period effect was +8.63 pp (11.6 pp in 2021 and 5.65 pp in 2022).

*Escherichia coli* also showed higher-than-expected resistance, with a mean COVID-period effect of +9.00 pp (+8.97 pp in 2021 and +9.03 pp in 2022). For *Proteus mirabilis*, resistance increased above the counterfactual by a mean of +4.41 pp (+4.14 pp in 2021 and +4.69 pp in 2022). The solid lines in Figure 4 represent observed resistance proportions over time, while the dashed lines indicate modeled counterfactual trends based on pre-COVID data. Divergence between the lines reflects the estimated COVID-period impact on resistance.

**Figure 5:**
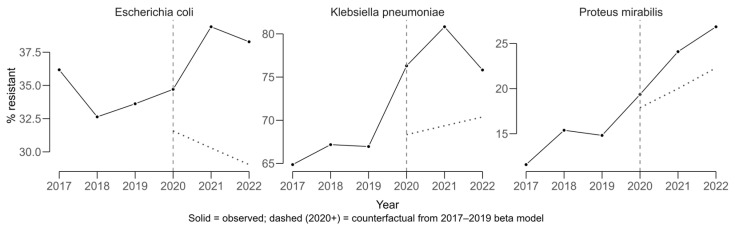
COVID-period effect analysis in trends for high-level aminoglycoside resistance (HLAR) and vancomycin resistance among *Enterococcus faecalis* and *Enterococcus faecium* (2017–2022).

The effects of the COVID period varied among different species of enterococci (Figure 5). High-level gentamicin resistance in *E. faecalis* was higher than expected, with a mean COVID-period deviation of +10.3 percentage points (pp) (+9.66 pp in 2021; +10.9 pp in 2022), whereas *E. faecium* showed a lower-than-expected HLAR, averaging −10.7 pp (−11.5 pp in 2021; −9.97 pp in 2022).

By contrast, vancomycin resistance in *E. faecalis* remained essentially unchanged relative to the counterfactual (mean −0.68 pp; −0.70 pp in 2021; −0.66 pp in 2022). In *E. faecium*, vancomycin resistance exceeded the counterfactual, with a mean of +7.37 pp (+1.82 pp in 2021; +12.9 pp in 2022), indicating a post-COVID rise above pre-pandemic trends.

### 2.5. Overall Resistance Patterns Across Microorganism–Antibiotic Combinations

To provide a comprehensive view of antimicrobial resistance (AMR) across the study period, researchers aggregated susceptibility test results by microorganism–antibiotic combination, pooling data from all years. For each combination, the total number of isolates tested, the number of resistant isolates, and the proportion of resistant isolates were calculated.

**Figure 6:**
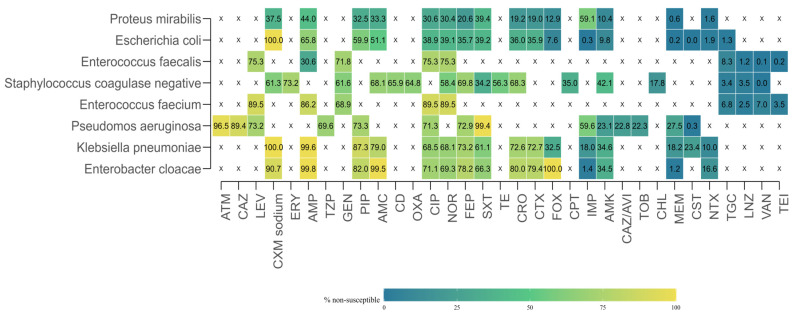
Overall antimicrobial resistance heatmap across microorganism–antibiotic combinations, 2017–2022. Color scale indicates the proportion of resistant isolates, with blue representing low resistance and yellow representing high resistance. White cells with an “X” indicate antibiotics that were not tested for the corresponding organism due to intrinsic resistance, clinical irrelevance, or local laboratory testing protocols, and should not be interpreted as missing data.

Marked heterogeneity in resistance rates was observed (Figure 6). Several organism–drug combinations displayed extremely high resistance levels, in some cases approaching or reaching 100%. Similarly, *Pseudomonas aeruginosa* exhibited high resistance to aztreonam (96.5%), and *Enterococcus faecium* to ampicillin (86.2%). In contrast, very low resistance rates were recorded for carbapenems in most *Enterobacterales species*. For example, resistance in *E. coli* was 0.26% to imipenem and 0.23% to meropenem, while *Enterobacter cloacae* showed resistance rates of 1.42% to imipenem and 1.21% to meropenem. Low resistance levels were also documented for glycopeptides in *Enterococcus* spp., with vancomycin resistance of 0.07% for *E. faecalis* and 7.01% for *E. faecium*.

Data availability was highly variable across combinations. In some cases, antimicrobials were not tested at all against specific organisms, either due to intrinsic resistance, clinical irrelevance, or local laboratory testing protocols. For example, macrolides such as erythromycin were absent from testing against Gram-negative bacilli, while several β-lactams were not reported for *Enterococcus* spp. This uneven distribution of testing is reflected in the heatmap (Figure 5), where white cells with an “X” indicate missing or insufficient data.

## 3. Discussion

The present study provides up-to-date data on the etiology and antimicrobial resistance of urine isolates from hospitalized patients in our tertiary care hospital during the period 2017–2022. The most significant findings are the dominant share of *E. coli* and the stable presence of *E. faecalis*, the increase in the isolation of *Candida* spp. in recent years, as well as the distinct increase in resistance to broad-spectrum antibiotics. These results are consistent with those described in a number of European and international studies, but also show specific local features that deserve discussion. In the following subsections, we discuss the etiological structure, the dynamics of resistance, and the possible factors that influence these trends [31].

### 3.1. Etiological Spectrum of Uropathogens

Our results on the etiological structure and antibiotic resistance in hospitalized patients with urinary tract infections confirm and correspond to the trends described in the literature but also demonstrate some worrying features at the local level. *E. coli* remains the dominant causative agent (33–49% in different years), which is consistent with data from the European Association of Urology (EAU) and other authors, who report a frequency between 50% and 70% in uncomplicated infections [15,32,33]. In alignment with our findings, *Escherichia coli* consistently emerges as the predominant uropathogen across European surveillance data, accounting for roughly 70% of urinary bacterial isolates [34]. Initially, M. Bader argued that in the setting of UTIs, the etiology and susceptibility of the causative agent are unpredictable [32], but later he found that for the successful management of UTIs, it is useful to think of infections caused by multidrug-resistant pathogens [35]. Therefore, when infection is suspected, patients should be referred for microbiological analysis of urine culture and antibiotic susceptibility testing of the isolated pathogen [32]. Although the benefits to patients of the use of antibiotics are clear, their overuse and misuse have contributed to the growing problem of resistance among pathogenic bacteria, which is a serious threat to public health [16].

Another representative causative agent of urinary tract infections is *Klebsiella pneumoniae*. *K. pneumoniae* and *P. mirabilis* were the second and third most common throughout the study period, with the frequency of *K. pneumoniae* (8.9–14.9%) being higher than some international observations, which may be related to nosocomial spread and pressure from antimicrobial therapy [36]. The increased isolation of fungi (*Candida* spp.) to 6% in 2022 is consistent with publications linking this finding to increased antibiotic use during the COVID-19 pandemic [37].

### 3.2. Antimicrobial Resistance Trends

The observed increase in ESBL-producing *E. coli* (from 34% to 38%) and *K. pneumoniae* (from 66% to 77%) is higher than the data of the study, where the prevalence of *E. coli* is about 20% in Europe [38]. This emphasizes that there must be stricter implementation of an antibiotic stewardship program. A similar upward trend has been described in other Bulgarian hospitals [36], suggesting that the problem is at the national level.

The study data demonstrate an increase in carbapenem resistance (KPC) in *K. pneumoniae* (from ~2% to over 40% for imipenem and meropenem), which significantly exceeds the values reported in the EU literature (about 7.5% according to ECDC, 2022). Such values are reported in countries in Eastern Europe, where cases of KPC producers have been identified [32].

Quinolone resistance in *K. pneumoniae* and *P. mirabilis* increased by over 10%, while in *E. coli*, there was a slight decrease. Similar heterogeneity was described by Xu et al. in 2025 [39], who linked the differences to local therapeutic practices and the frequency of prior treatment. Notably, resistance rates to fluoroquinolones and third-generation cephalosporins vary widely across the WHO European Region—fluoroquinolone resistance in *E. coli* exceeds 50% in several southern and eastern European countries, while third-generation cephalosporin resistance exceeds this threshold in nearly 10% of reporting nations [34].

The limited therapeutic options for *Enterococcus* spp. are of concern, with the increase in vancomycin-resistant (VRE) *E. faecium* reaching 8.9%. At the same time, the HLAR in *E. faecalis* (70%) remains stable. These values are close to those reported in Germany and other European countries (7–10%) [40].

### 3.3. Impact of the COVID-19 Pandemic

Our interrupted time series analysis showed a clear increase in resistance above the expected level for the period 2020–2022, especially in ESBL-producing *K. pneumoniae* and *E. coli*. This finding is supported by publications that link the pandemic to the wider use of broad-spectrum antibiotics and the weakening of control mechanisms in hospitals [40,41]. Moreover, the observed rise in carbapenem resistance in *K. pneumoniae* in our hospital exceeds that in *E. coli* and reflects observations from the EU/EEA and Southern/Eastern Europe, where high-risk clones such as ST258/512, ST101, and ST11 circulate [31].

Beyond Europe, studies from the Balkans highlight significantly elevated rates of multidrug-resistant UTIs, a trend possibly exacerbated by increased antibiotic use during the COVID-19 pandemic [42]. These observations underscore the critical need for ongoing, context-specific surveillance to inform both empirical therapy and stewardship initiatives.

### 3.4. Therapeutic Implications

Antibiotic treatment should be considered when there is clear clinical and laboratory evidence of urinary tract infection—such as significant bacteriuria accompanied by urinary symptoms—or in high-risk scenarios, including pregnancy, immunosuppression, or structural abnormalities of the urinary tract. The route of administration depends on the severity of the infection and the patient’s clinical condition: intravenous therapy is indicated for severe, complicated cases or when oral intake is not feasible, while oral regimens are generally preferred for stable, uncomplicated infections managed in outpatient settings [15].

In clinical decision-making, it is essential to account for local resistance patterns. To support this, microbiology laboratories in every healthcare facility should consistently report the circulating uropathogenic strains and their susceptibility profiles, enabling appropriate empiric therapy and antimicrobial stewardship [39]. Moreover, *Enterococcus species*—especially *Enterococcus faecalis*—have emerged as the second most frequently isolated uropathogen in certain populations (e.g., male outpatients, polymicrobial infections), sometimes accounting for 16% of cases [40]. This trend underscores the need for empiric regimens or local guidelines that address *Enterococcus* spp. coverage, given its intrinsic and acquired resistance to commonly used agents [41].

Current international guidelines recommend specific first-line agents for uncomplicated UTIs, such as nitrofurantoin and fosfomycin, but trimethoprim–sulfamethoxazole is not applicable to our area due to reported high levels of resistance. In contrast, complicated UTIs or those caused by resistant organisms may require broader-spectrum agents, including beta-lactam/beta-lactamase inhibitor combinations, cephalosporins, or fluoroquinolones, with the choice guided by urine culture and susceptibility results. These recommendations are supported by evidence-based resources such as the European Association of Urology (EAU) Guidelines on Urological Infections, which emphasize targeted therapy, optimal treatment duration, and antimicrobial stewardship to reduce the development of resistance [43].

Given the high local incidence of ESBL among *E. coli* (34–38%) and *K. pneumoniae* (66–77%), as well as carbapenem-resistant *K. pneumoniae* up to 40.6%, we recommend in hospitalized patients with systemic UTI empirical meropenem coverage against ESBL (meropenem), with early de-escalation according to an antibiogram; fluoroquinolones are not suitable for empirical use in local resistance of 38%. In critically ill patients with sepsis, management includes meropenem ± amikacin and urgent source control; in documented MDR/CRE infections, targeted therapy with ceftazidime/avibactam, meropenem/vaborbactam, or cefiderocol should be administered according to [43].

### 3.5. Strengths and Limitations

The present study has several strengths that increase the reliability of its findings. The large number of urine specimens processed over a six-year period and the substantial number of isolates provide robust statistical power to detect temporal shifts in both the etiological spectrum and antimicrobial resistance. The fact that all isolates were obtained and processed in the region’s largest tertiary care microbiology reference center ensures consistency of diagnostic methods and adherence to EUCAST standards across the study period. The use of advanced laboratory techniques, including MALDI-TOF and broth microdilution for colistin, enhances the accuracy of pathogen identification and susceptibility testing. Furthermore, the application of beta regression for bounded resistance proportions and interrupted time-series modeling for the COVID-19 period demonstrates methodological rigor and strengthens the interpretation of temporal changes.

Nevertheless, important limitations need to be acknowledged when interpreting the results. Because this is a retrospective, single-center study, the findings may not be fully generalizable to other hospitals in Bulgaria or to the community setting, where the case mix, diagnostic practices, and antibiotic pressures differ substantially. The study population consisted mainly of hospitalized patients, who are likely to overrepresent complicated infections, catheter-associated cases, and patients with repeated exposure to broad-spectrum antibiotics. This inevitably inflates resistance estimates compared to those observed in community-acquired urinary tract infections and introduces selection bias.

Moreover, the retrospective design meant that no patient-level clinical information was available, such as comorbidities, prior hospitalizations, device use, or previous antibiotic treatment. The absence of such variables precludes adjustment for important confounders that strongly influence the risk of resistant infections. In addition, detailed information on treatment regimens was not available in our dataset. As a result, antibiotic use could only be discussed in general terms, drawing on clinical guidelines and contextual interpretation rather than on direct prescribing data. This limits our ability to link observed resistance dynamics with prescribing practices and highlights the need for future studies that integrate microbiological findings with patient-level treatment data.

Temporal comparisons may also have been affected by changes in diagnostic practices. During the study period, MALDI-TOF was introduced into routine diagnostics, which improved species identification and could partly explain the apparent increase in less common pathogens and non-albicans Candida. Breakpoint revisions by EUCAST and the combination of different susceptibility testing methods could also contribute to apparent step changes in resistance proportions independent of underlying epidemiology. The use of cefotaxime resistance as a proxy for ESBL production, while pragmatic, is not fully specific, and molecular confirmation was not performed, limiting insights into clonal spread or the distribution of resistance genes.

Similarly, while the interrupted time-series framework highlights excess resistance during the COVID-19 period, it cannot fully disentangle the effect of the pandemic from other unmeasured factors such as changes in hospital admission patterns, antibiotic stewardship policies, or shifts in testing thresholds.

A further limitation of our study is the absence of ward-level and patient-level clinical information, as GDPR regulations require aggregated data extraction without unit-specific identifiers. Consequently, we were unable to perform analyses stratified by medical, surgical, pediatric, or high-dependency wards, or to differentiate between community- and healthcare-associated infections, catheter-related versus non-catheter-related UTIs, or complicated versus uncomplicated cases. Variables such as urinary catheter days were also unavailable, which prevented the calculation of CAUTI incidence rates. We fully recognize that these data would enrich the clinical interpretation of our findings. Nevertheless, our large-scale, laboratory-based surveillance still provides critical evidence on etiological spectrum and resistance trends in the region’s main referral center, which is essential for guiding empiric therapy, informing antimicrobial stewardship, and framing priorities for future multicenter, prospective studies that integrate microbiological and clinical datasets. Our study should therefore be understood as a surveillance resource rather than a clinical outcomes analysis.

Finally, the absence of incidence-based denominators, such as resistant isolates per 1000 patient-days or stratification by ward type, means that increases in resistance may partly reflect changes in the intensity of culturing rather than true epidemiological shifts.

## 4. Materials and Methods

### 4.1. Study Design and Setting

A retrospective, single-center epidemiological study was conducted at the Laboratory of Microbiology, University Hospital St. George in Plovdiv, Bulgaria, a tertiary care institution serving as a regional reference facility for microbiological diagnostics in Southeastern Bulgaria. The study period extended from 1 January 2017 to 31 December 2022. Inpatient samples were included to capture the spectrum of community-acquired and healthcare-associated UTIs.

### 4.2. Population, Eligibility, and Unit of Analysis

Urine specimens were submitted for microbiological analysis at the discretion of the treating physician when urinary tract infection was clinically suspected, with indications such as fever of unknown origin, urinary symptoms (dysuria, frequency, urgency, suprapubic pain), suspected pyelonephritis, sepsis of urinary origin, or preoperative evaluation. For statistical analyses, only the first isolate per patient per calendar year was retained to reduce correlation from repeat sampling, and polymicrobial cultures were analyzed at the organism level with per-patient de-duplication applied.

### 4.3. Sample Collection

During the study period from January 2017 to December 2022, the microbiology laboratory received 77,621 midstream self-catch urine samples from 25,087 hospitalized patients. Due to GDPR regulations, ward- or unit-level identifiers (medical, surgical, pediatric, or high-dependency units) were not available, and analyses were restricted to aggregated hospital-level data.

In this study, a UTI was defined as the presence of significant bacteriuria at thresholds consistent with clinical presentation, in accordance with EUCAST and BAM guidelines, together with clinical suspicion of infection. Specifically, symptomatic infections were considered when bacteriuria was ≥10^2^–10^3^ CFU/mL for typical uropathogens in concordant clinical settings (e.g., *E. coli*, *Staphylococcus saprophyticus* in women) and ≥10^3^ CFU/mL in men or catheterized specimens. For asymptomatic bacteriuria, ≥10^5^ CFU/mL of the same organism was required in two consecutive samples for women or in one sample for men. Polymicrobial growth without a dominant organism was interpreted as probable contamination and was excluded.

### 4.4. Urine Culturing

The samples underwent standard bacterial growth screening using the HB&L UROQUATTRO system (Alifax, Polverara, Italy). The urine samples were inoculated in a broth-containing vial (URO-QUICK Screening Kit, Alifax, Italy) following the manufacturer’s instructions. The screening time was set at 3 h.

Screen-positive urine samples were cultured using a calibrated loop of 1 μL (0.001 mL) and the streak method for semi-quantitative isolation on 5% sheep blood agar (HiMedia Laboratories, Maharashtra, India) and Eosin-Methylene Blue (EMB) medium (HiMedia Laboratories, India); for yeasts, Sabouraud-dextrose or chromogenic agar was used for *Candida* spp. (HiMedia Laboratories, India). The plates were incubated aerobically at 35–37 °C for 18–24 h. Yeast cultures were incubated at 30–35 °C for 24–48 h.

#### 4.4.1. Quantitation (CFU/mL)

Colonies were counted in the primary streak zone and converted to CFU/mL = colony count × (1/loop volume in mL), i.e., multiplied by 10^3^ for a 1 μL loop.

#### 4.4.2. Significant Bacteriuria—Thresholds and Interpretation

A single threshold of 10^5^ CFU/mL is not universally applied; clinical context and specimen type determine significance: 1. Asymptomatic bacteriuria (midstream): ≥10^5^ CFU/mL of the same organism in two consecutive specimens (women) or in a single specimen (men). 2. Symptomatic uncomplicated cystitis (women): Counts as low as ≥10^2^–10^3^ CFU/mL may be significant (e.g., for *E. coli*, *Staphylococcus saprophyticus*) when clinically concordant. 3. Symptomatic men: Counts ≥10^3^ CFU/mL are generally considered significant. 4. Catheterized urine/suprapubic aspiration: Counts ≥10^2^–10^3^ CFU/mL are typically considered significant in the appropriate clinical setting. 5. *Enterococcus* spp. and *Staphylococcus* spp.: Lower thresholds may be accepted when growth is pure and fits the presentation.

When ≥2 distinct morphotypes were present without a dominant organism and at low counts (<10^4^–10^5^ CFU/mL), results were interpreted as probable contamination, and a repeat specimen was recommended. If a single dominant organism exceeded the relevant threshold with a minor secondary population, the dominant isolate was reported.

Identification after isolation was done using standard biochemical methods (Remel, BioMérieux, Marcy-l’Étoile, France) and automated methods such as MALDI-TOF Vitek MS (BioMérieux, France) and Vitek 2 Compact (BioMérieux, France).

### 4.5. Antimicrobial Susceptibility Test

Antimicrobial susceptibility was tested using the Kirby–Bauer disk diffusion method (DDM), minimal inhibitory concentration (MIC), and an automated system (Vitek 2 Compact), and the results were interpreted according to the EUCAST standards for the corresponding year. CLSI standards were not applied in this study.

1. *Kirby–Bauer Disk Diffusion Method (DDM).* A 0.5 McFarland suspension was prepared from colonies incubated for 18–24 h. in sterile saline and verified by densitometer. Müller-Hinton agar and Müller–Hinton agar supplemented with 5% defibrinated horse blood and β-NAD were used according to EUCAST recommendations. Plates were surface-inoculated by swabbing in three directions to ensure confluent growth; antimicrobial disks were applied within 15 min of inoculation with adequate spacing to avoid overlapping zones. The plates were incubated aerobically at 35–37 °C for 18–24 h. Inhibition zone diameters were measured to the nearest 1 mm with a caliper and interpreted using EUCAST zone diameter breakpoints for the relevant year.
2. *Minimal Inhibitory Concentration.* Gradient diffusion strips (e.g., Etest) were applied to MH for selected organism–drug pairs or in cases where DDM results were borderline or discordant. Plates were incubated as above, and MICs were read at the ellipse–scale intersection and interpreted with EUCAST MIC breakpoints for the corresponding year.
3. *Broth microdilution (BMD).* When required by EUCAST (e.g., colistin) or for confirmatory testing, reference BMD in cation-adjusted MH broth (no surfactants) was performed following the manufacturer’s instructions and interpreted per EUCAST; gradient diffusion or automated methods were not used for colistin.
4. *Vitek 2 Compact—AST* inoculum and cards. A 0.50–0.63 McFarland suspension in 0.45% saline was prepared from fresh colonies and inoculated into organism-appropriate AST cards (e.g., AST-N for Gram-negatives, AST-P for Gram-positives), following the manufacturer’s instructions.

The results were reported using EUCAST S/I/R categories, applying urine-specific breakpoints where defined.

### 4.6. Data Management and Quality Control

Microbiological and antimicrobial susceptibility data were extracted in aggregated format from the laboratory information system, where each row represented a microorganism–antibiotic combination, the total number of isolates tested, and the number classified as resistant. Aggregated data were structured to preserve the temporal dimension (year of isolation) and organism-level detail while ensuring patient confidentiality.

Data cleaning procedures included the removal of duplicates, exclusion of non-bacterial isolates where relevant to statistical models, and verification of species identification against internal quality control standards. Resistance proportions were calculated as the number of resistant isolates divided by the total number of isolates tested for each microorganism–antibiotic combination.

### 4.7. Statistical Analysis

Analyses were performed using R statistical software (version 5.1, R Foundation for Statistical Computing, Vienna, Austria; https://www.r-project.org/, accessed on 28 August 2025). Descriptive epidemiology was performed prior to modeling. Counts and proportions were reported for organism distribution overall and by year, and for resistance by organism–antibiotic combination. Then, 95% confidence intervals (CIs) for binomial proportions were calculated using either the Exact (Clopper–Pearson) method. Between-year differences in proportions were screened with χ^2^ tests or Fisher’s exact tests where appropriate, and linear-by-linear association (trend) tests were applied to evaluate ordered annual trends in crude proportions.

Temporal trends in annual resistance proportions were estimated using beta regression with a logit link (package *betareg*, version 3.2-4, accessed on 28 August 2025) [44], with calendar year modeled as a continuous predictor. Beta regression was selected because the outcome (proportion resistant) is bounded in (0.1) and typically heteroscedastic; zero–one inflation was handled by standard proportional adjustments where needed [45]. Effects were expressed as annual percentage point (pp) changes with 95% CIs, derived from marginal effects on the response scale.

Potential pandemic-related deviations were assessed using an Interrupted Time Series (ITS) framework. Pre-pandemic observations (2017–2019) were used to fit counterfactual beta-regression trajectories; observed values for 2020–2022 were then contrasted with counterfactual predictions to estimate absolute COVID-period effects in pp for key organism–antibiotic endpoints (e.g., ESBL proxy, quinolones, carbapenems) [46]. Model diagnostics comprised residual checks, assessment of leverage, and sensitivity to pre-period length. Before–after contrasts between the first and last study years were also conducted using Fisher’s exact tests. Effect sizes were reported as absolute risk differences (pp) with 95% CIs. Where any cell count was zero, the Haldane–Anscombe correction was applied for ratio measures reported in sensitivity analyses [47].

Familywise multiplicity arising from organism–antibiotic panels and multiple endpoints was addressed by controlling the false discovery rate (FDR) using the Benjamini–Hochberg procedure; adjusted *p*-values < 0.05 were considered statistically significant [48].

## 5. Conclusions

In a six-year inpatient follow-up from a Bulgarian tertiary care center, *E. coli* remained the dominant uropathogen. Simultaneously, the increase in candiduria reflected a stable bacterial burden and suggested a trend towards rising fungal isolation. The resistance trends are clinically relevant: ESBL increased in the most common *Enterobacterales species*, fluoroquinolone resistance varied among the dominant species, and carbapenem resistance increased in *K. pneumoniae* by about 40%, but remained less common in *E. coli*. These data support locally adapted empirical therapy regimens that reduce the importance of fluoroquinolones and prioritize ESBL-active β-lactams in systemic/complicated urinary tract infections, with early de-escalation recommended after recovery of susceptibility. Given the level of carbapenem-resistant *K. pneumoniae*, enhanced infection management, prevention measures, as well as molecular typing are needed to track resistotypes. Future work should include multicenter, prospective cohorts linking rapid diagnosis to outcomes to optimize therapy. Our results confirm the need for locally adapted empirical regimens based on current resistance data; application of rapid methods for identification and antibiotic susceptibility (mPCR, MALDI-TOF, HB&L Uroquattro); and strengthening antimicrobial control and monitoring, especially regarding carbapenem-resistant enterobacteria.

## Figures and Tables

**Table 1:** Distribution of urinary isolates by year (2017–2022) at the Microbiology Laboratory, University Hospital St. George, Plovdiv, Bulgaria ^1^.

Year	Number of Isolates	Relative Share (%)	Gram-Negative (%)	Gram-Positive (%)	Fungi (%)
2017	1711	14.3	73.12	24.9	1.99
2018	1323	10.5	72.94	24.26	2.8
2019	1204	9	78.24	19.27	2.49
2020	1172	10.9	76.02	21.33	2.65
2021	1535	13.8	69.58	25.93	4.5
2022	1972	13.6	69.78	24.44	5.78

^1^ Data represent urine isolates from both inpatient samples processed between 2017 and 2022. Relative share (%) indicates the proportion of positive isolates out of all urine samples submitted for microbiological testing in the respective year.

## Data Availability

The original contributions presented in this study are included in the article. Further inquiries can be directed to the corresponding author.

## Abbreviations

UTI/UTIs	Urinary Tract Infection(s)
HB&L	High Bacterial Load
MALDI-TOF MS	Matrix-Assisted Laser Desorption/Ionization Time-of-Flight Mass Spectrometry
EMB	Eosin-Methylene Blue
MIC	Minimum Inhibitory Concentration
AMR	Antimicrobial Resistance
EUCAST	European Committee on Antimicrobial Susceptibility Testing
ESBLs	Extended-Spectrum Beta-Lactamases
HLAR	High-Level Aminoglycoside Resistance
COVID-19	Coronavirus Disease 2019
LUTI	Lower Urinary Tract Infection
BAM	Bulgarian Association of Microbiologists
CFU/ml	Colony Forming Units per milliliter
DDM	Disk Diffusion Method
mPCR	Multiplex Polymerase Chain Reaction
PCR	Polymerase Chain Reaction
R	R Statistical Software
ECDC	European Centre for Disease Prevention and Control
WHO	World Health Organization
EAU	European Association of Urology
ESKAPE	*Enterococcus faecium*, *Staphylococcus aureus*, *Klebsiella pneumoniae*, *Acinetobacter baumannii*, *Pseudomonas aeruginosa*, *Enterobacter* spp.
EU/EEA	European Union/European Economic Area.
MDR	Multidrug-Resistant
CRE	Carbapenem-Resistant Enterobacteriaceae
μL	Microliter
mm	Millimeter
Etest	Epsilometer Test
MH	Mueller–Hinton Agar
BMD	Broth microdilution
S/I/R	Susceptible/Intermediate/Resistant
CI	Confidence Interval
χ^2^	Chi-Squared Test
Pp	Percentage Point
ITS	Interrupted Time Series
FDR	False Discovery Rate
